# Combination of Cyclopamine and Tamoxifen Promotes Survival and Migration of MCF-7 Breast Cancer Cells – Interaction of Hedgehog-Gli and Estrogen Receptor Signaling Pathways

**DOI:** 10.1371/journal.pone.0114510

**Published:** 2014-12-12

**Authors:** Maja Sabol, Diana Trnski, Zvonimir Uzarevic, Petar Ozretic, Vesna Musani, Maja Rafaj, Mario Cindric, Sonja Levanat

**Affiliations:** 1 Division of Molecular Medicine, Rudjer Boskovic Institute, Zagreb, Croatia; 2 Faculty of Education, Josip Juraj Strossmayer University of Osijek, Osijek, Croatia; University of Alabama at Birmingham, United States of America

## Abstract

Hedgehog-Gli (Hh-Gli) signaling pathway is one of the new molecular targets found upregulated in breast tumors. Estrogen receptor alpha (ERα) signaling has a key role in the development of hormone-dependent breast cancer. We aimed to investigate the effects of inhibiting both pathways simultaneously on breast cancer cell survival and the potential interactions between these two signaling pathways. ER-positive MCF-7 cells show decreased viability after treatment with cyclopamine, a Hh-Gli pathway inhibitor, as well as after tamoxifen (an ERα inhibitor) treatment. Simultaneous treatment with cyclopamine and tamoxifen on the other hand, causes short-term survival of cells, and increased migration. We found upregulated Hh-Gli signaling under these conditions and protein profiling revealed increased expression of proteins involved in cell proliferation and migration. Therefore, even though Hh-Gli signaling seems to be a good potential target for breast cancer therapy, caution must be advised, especially when combining therapies. In addition, we also show a potential direct interaction between the Shh protein and ERα in MCF-7 cells. Our data suggest that the Shh protein is able to activate ERα independently of the canonical Hh-Gli signaling pathway. Therefore, this may present an additional boost for ER-positive cells that express Shh, even in the absence of estrogen.

## Introduction

Breast cancer is a heterogeneous disease divided into three major subtypes with differing response to therapy: the hormone receptor-positive (with either estrogen receptor (ER) or progesterone receptor (PR) expression), the HER-2 amplified, and the triple-negative cancer (ER-negative, PR-negative and HER2-negative). For ER-positive tumors, therapy is mostly based on inhibition of estrogen synthesis or inhibition of estrogen receptor activity, for example tamoxifen is commonly used. However, many of the patients do not respond to endocrine treatment or develop acquired resistance [1].

The Hedgehog-Gli (Hh-Gli) signaling pathway is involved in embryonic development of mammary buds [2], and the pathway genes are expressed in the mammary gland during postnatal development [3]. Aberrant activation of the pathway is associated with tumorigenesis and developmental malformations. The pathway is initiated with binding of the ligand Hedgehog (Sonic, Indian or Desert Hh) to its transmembrane receptor Patched (Ptch). Ptch relieves its repression of Smoothened (Smo), causing a phosphorylation cascade and the release of transcription factor Gli from Suppressor of Fused (SuFu). Gli translocates to the nucleus, where it initiates target gene transcription. Hh-Gli pathway target genes are involved in proliferation and differentiation, cell survival, self-renewal, angiogenesis, and pathway autoregulation [4]–[6].

Hh-Gli signaling pathway hyperactivation has previously been detected in breast tumors [7]–[9]. *PTCH1* gene was found downregulated due to promoter hypermethylation [10], [11]. *SHH* promoter is frequently hypermethylated in the normal breast and this methylation is lost in breast tumors [12]. *SHH* is one of the signature genes associated with poor prognosis of inflammatory breast cancer [13]. Mutations in *PTCH1*, *SMO* and *SHH* genes have been examined in breast cancer: some studies found mutations [14], [15], while others did not [16], [17]. However, biallelic Pro1315Leu (C3944 T) *PTCH1* polymorphism was found associated with breast cancer risk when combined with oral contraception [18]. Loss of heterozygosity of the *PTCH1* gene is found in 30% of breast cancer patients [10]. The effects of cyclopamine, a Hh-Gli pathway inhibitor, on breast cancer have already been addressed in several studies. It was shown to cause growth inhibition mediated by apoptosis of some breast cancer cell lines [7], [19], while cells derived from normal breast tissue are not responsive to cyclopamine [20]. The Hh-Gli signaling pathway has been implicated in tamoxifen resistance. It was shown that a small molecule SMO inhibitor GDC-0449 can improve the outcome of tamoxifen-resistant tumors. Addition of tamoxifen to GDC-0449 had additional benefits *in vitro* but not *in vivo* [21]. Recently, cyclopamine was shown to have anti-proliferative, anti-invasive and anti-estrogenic potency in human breast cancer cells by suppressing the MAPK/ERK signaling pathway. Cyclopamine decreased ERα protein levels in MCF-7 cells and the authors speculate that combining cyclopamine with anti-estrogen therapies could lower the doses and side-effects [22].

Here we show a surprising, unfavorable effect of combined inhibition of Hh-Gli signaling and ERα in human ER-positive breast cancer cells and the potential underlying mechanism. In addition we also show a new, non-canonical interaction between the Hh-Gli and ERα signaling pathways.

## Materials and Methods

### Cell culture experiments

MCF-7 (ATCC, HTB-22) and SkBr-3 (ATCC, HTB-30) breast cancer cell lines were a kind gift from Dr. Sanja Kapitanović. Both cell lines were maintained in DMEM supplemented with 10% fetal bovine serum (FBS) and were mycoplasma-free.

MTT assay: cells were plated in 96-well plates 24 hours before treatment, in quadruplicates for each tested concentration: cyclopamine 0.5–7.5 µM (Toronto Research Chemicals, Toronto, Ontario, Canada), tamoxifen 1–10 µM (Toronto Research Chemicals). Combined treatments were with either cyclopamine for 48 h followed by tamoxifen for 48 h, tamoxifen for 48 h followed by cyclopamine for 48 h, cyclopamine + tamoxifen simultaneously for 48 h, cyclopamine + tamoxifen simultaneously for 96 h. Competition experiments: compounds were added simultaneously and MTT assay was performed after 48 h.

Gene expression studies: cells were plated into 6-wells in duplicates 24 h before treatment, and treated with cyclopamine (2.5 µM), Shh protein (3 ng/µl, kind gift from Dr. Anna Kenney) and tamoxifen (1 µM for MCF-7, which is the LD50 dose, or 5 µM for SkBr-3 (LD50 was not reached for SKBr-3, therefore a higher dose was used)) for 24 h or cyclopamine + tamoxifen for 48 and 96 h.

Transfection experiments: cells were transfected with 1 µg of pcDNA4nlSMtGLI1 plasmid expressing the Gli1 transcription factor (kind gift from Dr. Fritz Aberger) using Lipofectamine reagent (Life Technologies, Carlsbad, California, USA). Medium was changed after 5 h and specified wells were treated with Shh protein (3 ng/µl); cells were collected 48 h later.

*PTCH1* silencing: cells were transfected with 50 nM Silencer Select siRNA (Life Technologies, s11442) or Silencer Negative Control #1 siRNA (Life Technologies) using siPORT NeoFX (Life Technologies) transfection reagent. Medium was changed after 24 h, and cells were collected after 24 or 48 h.

### Wound healing assay

MCF-7 cells were grown to confluence in 24-well plates and serum starved over night. The following day monolayers were wounded with a plastic 200 µl pipette tip and washed with medium to remove detached cells. The wounds were allowed to close in medium without any treatment or in the presence of 10 µM cyclopamine, 10 µM tamoxifen or both drugs together. Images were taken at the 0 and 26 h time points. The wounds were photographed at 10x magnification, on the Olympus CKX41 inverted microscope linked to an Olympus E330 camera (Olympus, Shinjuku, Tokyo, Japan). Images were analyzed using the TScratch software, developed by the Koumoutsakos group (CSE Lab), at ETH Zürich [23].

Each time point was normalized to the 0 h image area and reported as the percent of open wound area. For the comparison of open wound areas between different treatments a one-way ANOVA with Newman-Keuls post hoc test for multiple pairwise comparisons was used. Two-tailed p value less than 0.05 was considered statistically significant. Statistical analysis was performed with GraphPad Prism 6 for Windows, version 6.05 (GraphPad Software, San Diego, California, USA).

### Transwell migration assay

To assay the migration of cells, 5×10^4^ cells in 500 µl of serum-free medium were seeded onto 8-µm pore Transwell Inserts (Corning, Corning, NY) in the absence of any treatment or in the presence of 10 µM cyclopamine, 10 µM tamoxifen or a combination of cyclopamine and tamoxifen. The lower chambers were filled with 1 ml of complete medium. After 48 h the cells that had not migrated were wiped off the upper side of the filter using a cotton swab. Migrated cells were fixed with 4% paraformaldehyde/PBS for 10 minutes and subsequently stained with crystal violet for 1 h. Images of five independent fields per insert were taken at 20x magnification using the Olympus BX51 microscope, and the number of migrated cells was counted. For the comparison of the number of migrated cells between different treatments a one-way ANOVA with Newman-Keuls post hoc test for multiple pairwise comparisons was used.

### Quantitative real-time PCR (qRT-PCR)

RNA extraction and qRT-PCR were performed as previously described [24], with primers *ERα* F 5′-CAGATGGTCAGTGCCTTGTTGG-3′, R 5′-CCAAGAGCAAGTTAGGAGCAAACAG-3′ [25] and *RPLP0*, *PTCH1* and *GLI1* [26], [27]. Expression was normalized using *RPLP0* housekeeping gene and relative fold change was calculated using the 2^−ΔΔCt^ formula.

### Immunofluorescent staining

Immunofluorescent staining and confocal microscopy were performed as previously described [24]. The following primary antibodies diluted 1∶100 were used: rabbit polyclonal anti-Hh (Santa Cruz Biotechnology, Dallas, Texas, USA, sc-9024), mouse monoclonal anti-ERα (Santa Cruz Biotechnology, sc-8002). For quantification of nuclear staining, three visual fields of magnification 60–100x were examined and cells were counted (non-treated (NT) N = 79; Shh treatment N = 124). Quantification of nuclear staining was obtained by determining the percent of cells showing positive ERα nuclear staining. For colocalization analysis of Shh and ERα, confocal images were examined using the Manders’ coefficient plugin of the ImageJ software (v 1.45e) for colocalization of green and red signals (red N = 5; green N = 5) [28]. The difference in nuclear staining and co-localization between untreated samples and each treatment was tested using one-way ANOVA with Dunnett's post hoc multiple comparisons test.

### Co-Immunoprecipitation

For co-immunoprecipitation experiments Protein G Dynabeads (Life Technologies) were coated with 5 µg anti-ERα antibody per sample and cell lysates were immunoprecipitated as per manufacturer’s instructions (Invitrogen, Rev. 005). Dynabeads without bound antibody were used as negative control. Samples were eluted with 1x loading buffer and heated 10 min at 70°C before analysis on Western blot.

### Western blot

Fifty µg of protein (determined by Bio-Rad Protein Assay; Bio-Rad, Hercules, California, USA) was loaded on SDS-polyacrylamide gel, transferred to a nitrocellulose membrane and blocked in 5% milk. Primary antibodies (diluted 1∶250) for Shh and ERα were the same as for the immunofluorescence experiment, additionally goat polyclonal anti-Ptch1 (Santa Cruz Biotechnology, sc-6147) and rabbit polyclonal anti-Gli1 (Santa Cruz Biotechnology, sc-20687) were used. Actin (Santa Cruz Biotechnology, sc-1616, goat polyclonal, diluted 1∶500) was used as loading control. After washing, membranes were incubated with the appropriate secondary HRP-conjugated antibody (Santa Cruz Biotechnology). Proteins were visualized using Super Signal West Pico and Femto reagents (Thermo Fisher Scientific, Waltham, Massachusetts, USA).

### Proteomic profiling by 2D-gel electrophoresis and mass spectrometry

#### Sample preparation

Cells were seeded in four 10 cm dishes for each treatment. After 24 h cells were treated with a combination of 5 µM cyclopamine and 10 µM tamoxifen in culture medium without serum for 48 h. The cells were then harvested at 4000 g (Tehtnica, Centric 400, Železniki, Slovenia) for 6 min, washed five times in 10 mM tris (hydroxymethyl) aminomethane (Tris)-sorbitol buffer, pH 7 and lysed with TissueRuptor (Qiagen, Venlo, Netherlands). The DNA and RNA were removed after treatment with DNase I and RNase A. The reconstituted proteins were precipitated overnight at −20°C with ice-cold acetone and centrifuged for 20 min at 5000 g [29]. The proteins were resuspended in rehydration solution for isoelectric focusing (IEF) containing 7 M urea, 2 M thiourea, 4% 3-[(3-cholamidopropyl)-dimethylammonio]-1-propanesulfonate hydrate (CHAPS) and 1% dithiothreitol (DTT) (w/v). Protein concentration in solution was estimated with Bradford protein assay.

#### Two-dimensional electrophoresis

Immobilized pH gradient strips (IPG; 17 cm, non-linear, pH 3–10) were rehydrated for 14 h with 350 mL of rehydration solution containing 7 M urea, 2 M thiourea, 4% CHAPS, 1% DTT (w/v) and 1.5 mg/mL of total protein. The IEF was carried out with a Protean IEF Cell (Bio-Rad) with a low initial voltage and an applied voltage gradient up to 7000 V. The total V×t product applied was 90 000 Vh for each strip at 20°C. The strips were equilibrated in equilibration buffer containing 20 mM DTT, 50 mM Tris adjusted to pH 6.8, 6 M urea, 2% sodium dodecyl sulfate (SDS) (w/v), 30% glycerol (v/v) and 0.01% bromophenol blue (BPB) (w/v) on a tilt table for 15 min. The solution was discarded and the same equilibration buffer solution without the addition of DTT and with the addition of 25 mM iodoacetamide was used for a 15 min protein alkylation reaction. The strips were placed on a 1 mm thick 12% polyacrylamide gel and sealed with 0.1% (w/v) agarose in SDS-electrophoresis buffer containing 0.01% (w/v) BPB. In the second dimension, the electrophoresis was run for 1 h at 15 mA per gel and then at 20 mA for 600 Vh. The electrophoresis was terminated after 30 mA per gel until the BPB reached the bottom of the gel. Tris-glycine running buffer containing 25 mM Tris, 190 mM glycine and 0.1% (w/v) SDS was used in the second dimension. Obtained gels were stained with Coomassie brilliant blue (CBB) G-250 stain [30].

#### Differential display analysis

Differential display analysis of the gel data sets was undertaken by comparing images of control gel (non-treated cell cultures) with the gel of treated cells (combination of cyclopamine and tamoxifen). Densitometry analysis was performed with image analysis software (Discovery Series PDQuest 2-DE analysis software package version 7.4.0.) integrated with a VersaDoc 4000 Imaging System (Bio-Rad). Master gels were used to obtain the differences between protein profiles of non-terated and treated cell cultures.

#### In-gel digestion

Differentially displayed protein spots were excised from 2-DE gels into small pieces and subjected to in-gel digestion with trypsin according to Shevchenko et al [31].

#### Data analysis and protein identification

Samples were mixed with α-cyano-4-hydroxycinnamic acid 1∶5, v/v (5 mg/mL; Fluka, Switzerland) and spotted onto a metal plate. MS acquisition was performed with a 4800 Plus MALDI TOF/TOF analyzer (Applied Biosystems, Carlsbad, California, USA) equipped with a 200 Hz, 355 nm Nd:YAG laser. Ions were analyzed in reflectron mode using positive polarity. The instrument parameters were set using the 4000 Series Explorer software (version 3.5.3, Applied Biosystems). Mass spectra were obtained by averaging 1000 laser shots covering a mass range of m/z 900 to 4000. MS/MS of the 10 most intense precursor signals from MS spectra was achieved by 1 keV collision energy in positive ion mode with air as a collision gas and by averaging 1600 laser shots.

Data were analyzed using ProteinPilot (ProteinPilot^TM^ Software 4.5., 2012 AB SCIEX) [32] for searching against the NCBI database using the *Homo sapiens* taxonomy. The search parameters allowed for two missed cleavage, trypsin digestion with a peptide tolerance = 0.3 Da and MS/MS tolerance = 0.5 Da. Only significant scores (greater than 39, p<0.05) for the peptides defined by a Mascot probability analysis were considered to be confidently identified peptides/proteins.

## Results

### MCF-7 and SkBr-3 cells are responsive to cyclopamine and tamoxifen treatment – combination shows unusual adverse effects

**Figure 1:**
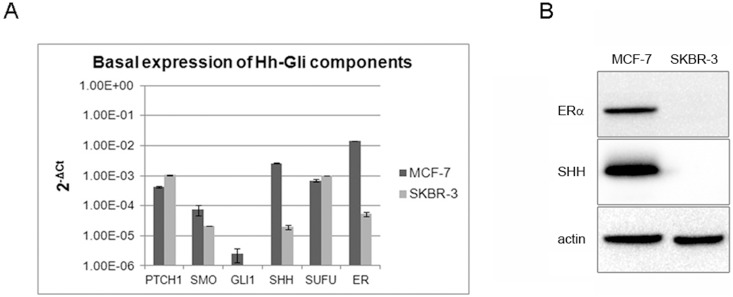
Basal gene expression levels of Hh-Gli pathway components and ERα in MCF-7 and SkBr-3 cell lines normalized relative to expression of the housekeeping gene *RPLP0* and shown as 2^−ΔCt^ values on logarithmic scale (A); Expression of ERα and SHH proteins in MCF-7 and SkBr-3 cell lines (B).

Both the ER-positive MCF-7 and the ER-negative SkBr-3 show expression of Hh-Gli pathway components. The major difference between the two cell lines was the expression of Shh and ERα, while the MCF-7 cell line expressed Shh and ERα both on gene and protein (Shh-N, 19 kDa) level, SkBr-3 cells showed low levels of *SHH* and *ERα* gene expression and no expression at protein level (Fig. 1A, B). SkBr-3 cells also showed no expression of *GLI1* (Fig. 1A).

**Figure 2:**
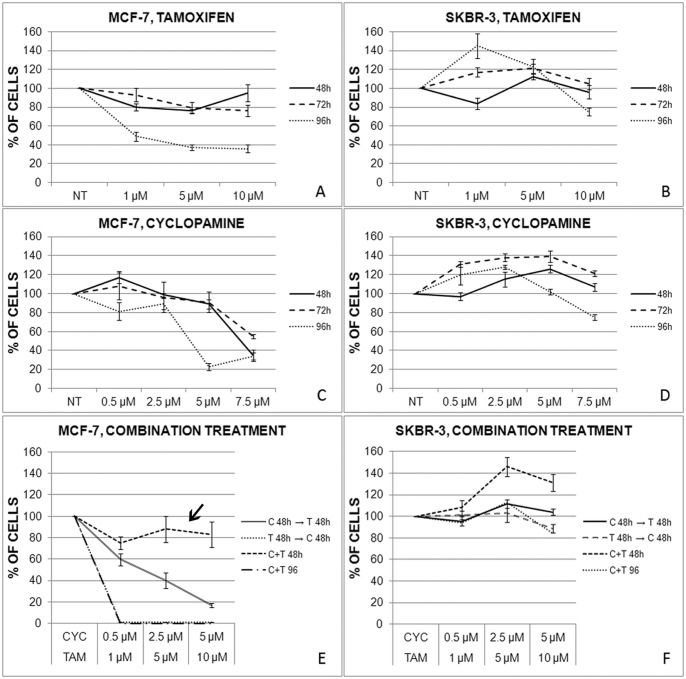
Cell viability after tamoxifen (A,B), cyclopamine (C,D) or combined treatment (E,F) in MCF-7 and SkBr-3 cell lines. Tamoxifen and cyclopamine each inhibit proliferation of MCF-7 cells in a dose dependent manner (A,C). When administered simultaneously, they cause a short term survival effect in MCF-7 cells (C+T 48 h) – pointed out with arrow, whereas long term simultaneous treatment induces strong cell death in these cells (C+T 96 h). Combination treatment of cyclopamine for 48 h followed by tamoxifen for 48 h (C 48 h →T 48 h) or vice versa (T 48 h →C 48 h) showed an effect similar to tamoxifen alone (E). Tamoxifen and cyclopamine show only a mild inhibitory effect on SkBr-3 cell proliferation at longest exposures (B,D) while combined treatment has no pronounced effect (F).

MCF-7 cells were responsive to both Hh-Gli signaling downregulation with cyclopamine, and ERα inhibition with tamoxifen, which both decreased MCF-7 cell proliferation. Both treatments had a significantly weaker effect on the ER-negative SkBr-3 cell line (Fig. 2A–D). To determine the effects of a combined treatment on cell proliferation, cells were treated with cyclopamine and tamoxifen in four different combinations: cyclopamine for 48 h followed by tamoxifen for 48 h, tamoxifen for 48 h followed by cyclopamine for 48 h, cyclopamine + tamoxifen simultaneously for 48 h and 96 h (Fig. 2E, F). In most cases, the combined effect was very similar to the effect of tamoxifen alone. However, a short-term combined treatment did not cause significantly decreased proliferation in MCF-7 cells (Fig. 2E).

**Figure 3:**
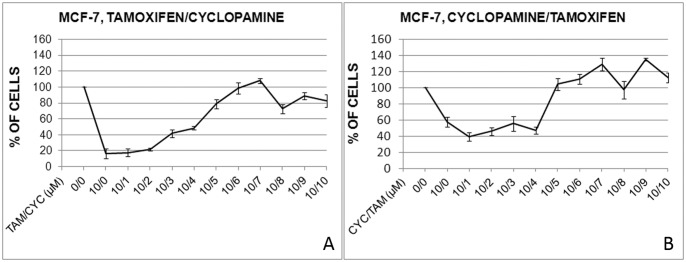
Effect of cyclopamine and tamoxifen combination on MCF-7 cell proliferation. When tamoxifen is in higher concentrations, and cyclopamine in lower concentrations, MCF-7 cell viability is decreased. However, when cyclopamine concentration is increased (with tamoxifen concentration remaining constant) cell viability increases (A). Similar effect can be seen vice-versa, when cyclopamine concentration is constant and tamoxifen concentration is increased (B) as measured by MTT assay after 48 h.

We tested the possible competition of cyclopamine and tamoxifen in both cell lines: cells were treated with a constant concentration of one compound, combined with a range of increasing concentrations of the second compound. For SkBr-3 cell line, there was no significant difference in compound activity (data not shown). In the MCF-7 cell line, however, increasing concentrations of the second compound increased short-term cell survival; regardless of the order of administration (Fig. 3). This suggests that even though cyclopamine and tamoxifen alone show inhibitory effects on MCF-7 cells, when administered together they counter each other’s effects.

### Combined cyclopamine and tamoxifen treatment alters Hh-Gli signaling pathway activity in MCF-7 cells and promotes cell migration

**Figure 4:**
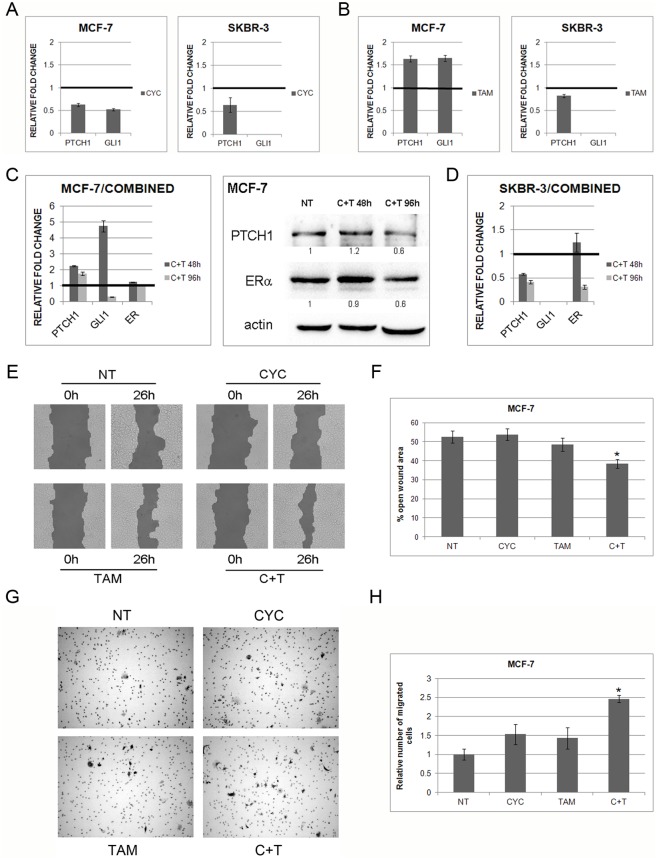
Effects of cyclopamine (A) and tamoxifen (B) on Hh-Gli pathway gene expression in MCF-7 and SkBr-3 cells. The Hh-Gli pathway is upregulated after short-term combined treatment in MCF-7, but the effect is negated after longer treatment. On the Western blot image, band quantification relative to actin and non-treated cells is denoted below the bands. (C). The effect of combined treatment on SkBr-3 cell line is weak (D). Gene expression levels are shown on graph as relative fold change relative to non-treated conditions with reference value 1 pointed out with emboldened bar. Only combined cyclopamine and tamoxifen treatment induces migration in MCF-7 cells. Representative images of the wound healing assay at 0 and 26 h (after processing with TScratch software [23]) are shown for non-treated conditions (NT; N = 16), cyclopamine treatment (CYC; N = 16), tamoxifen treatment (TAM; N = 14) and combined treatment with cyclopamine and tamoxifen (C+T; N = 12) (E). Quantitative analysis of the percentage of open wound areas is shown on the graph, (*) P<0.05 (F). Transwell migration assay confirmed increased migration capacity of cells after combined cyclopamine and tamoxifen treatment. Representative images of migrated cells after 48 h are shown for non-treated conditions (NT; N = 15), cyclopamine treatment (CYC; N = 15), tamoxifen treatment (TAM; N = 15) and combined treatment (C+T; N = 15) (G). Quantitative analysis of the relative number of migrated cells (analyzed relative to non-treated cells) is shown on graph, (*) P<0.0001 (H).

Prior to investigating the combined effect of cyclopamine and tamoxifen, we first tested the effect of cyclopamine and tamoxifen alone on the Hh-Gli signaling pathway. Both cell lines showed a similar response when treated with cyclopamine. 24 h after treatment with cyclopamine *PTCH1* and *GLI1* expression was downregulated in the MCF-7 cell line and *PTCH1* was downregulated in SkBr-3, suggesting pathway inhibition (Fig. 4A). Tamoxifen treatment upregulated *PTCH1* and *GLI1* expression in MCF-7 cells, while *PTCH1* levels remained unchanged in the SkBr-3 cell line (Fig. 4B).

Although some pathway components are expressed, the pathway shows a low level of activity in SkBr-3 cells, but with downregulation possibility with cyclopamine, which may be carried out through other pathway effectors such as Gli2 or Gli3 that were not tested in this study.

Combined treatment with cyclopamine and tamoxifen showed a different effect on ER-positive and ER-negative cell line. ER-positive MCF-7 cell line showed increased Hh-Gli signaling after short-term treatment. Even though the level of *PTCH1* mRNA was still elevated after long-term treatment, a decreasing tendency was visible compared with short-term treatment. This is confirmed by the level of Ptch1 protein, which was decreased 40% after long-term combined treatment compared with non-treated cells. ERα protein level showed no change after shorter treatment but declined after longer treatment (Fig. 4C). SkBr-3, however, showed generally downregulated Hh-Gli signaling after combined treatment regardless of treatment duration (Fig. 4D).

Wound induced migration assay was performed to test whether the combination of cyclopamine and tamoxifen has an effect on the ability of MCF-7 cells to migrate, in addition to the effects on Hh-Gli signaling and cell proliferation. Cyclopamine or tamoxifen alone had no effect on the wound closing rate, compared with the wound closing in the absence of any treatment. On the other hand, combined treatment with cyclopamine and tamoxifen accelerated the wound healing process compared with non-treated conditions and with cyclopamine or tamoxifen alone (Fig. 4E, F). To confirm the obtained results a transwell migration assay was performed. This assay confirmed no effect of either cyclopamine or tamoxifen alone on the migration rate when compared with the non-treated cell migration rate. It also confirmed a higher migration capacity of MCF-7 cells treated with a combination of cyclopamine and tamoxifen compared with non-treated cells or cells treated with cyclopamine or tamoxifen alone (Fig. 4G, H). The increase in the migration capacity was even higher when analyzed with the transwell migration assay in comparison with the wound healing assay.

### Proteomic profiling of cells treated with cyclopamine and tamoxifen versus non-treated cells

Differential protein expression analysis was conducted to identify the profile of expressed proteins in cells treated with a combination of cyclopamine and tamoxifen. These differentially expressed proteins may explain the effects of the combined treatment with cyclopamine and tamoxifen on cell proliferation and migration. The identified proteins are listed in Table 1. Images of the obtained 2-D gels are shown in S1 Figure. As opposed to cells treated with a combination of drugs, non-treated cells mostly show expression of proteins involved in response to topologically incorrect and unfolded proteins; carbohydrate and amino acid metabolism, gene transcription, RNA processing and translation. Interestingly, the heat shock protein 27 (HSP27) is expressed in both non-treated cells and those treated with a combination of cyclopamine and tamoxifen. However, the protein is shifted in the 2-D gel of treated cells compared with its localization in the 2-D gel of non-treated cells, which could indicate a posttranslational modification after treatment. Additionally, the GRP78 precursor protein, which is a known survival factor [33] that can mediate signaling pathways that lead to proliferation and invasion [33], [34] was expressed only in treated cells. Also, two proteins that can be linked with upregulation of proliferation and migration showed an increase in expression in treated cells, namely prohibitin and keratin 8 [35], [36]. Together these results indicate that certain proteins involved in tumor cell survival and migration are upregulated or possibly activated.

**Table 1:** Differentially expressed proteins in MCF-7 cells treated with cyclopamine and tamoxifen compared with non-treated control cells.

	**2-D gel of control MCF-7 cells**				
**No**	**Protein Description**		**GI Accession**	**Score**	**General Functions**
1	Heat shock protein 90-alpha		gi|32488	142	• Molecular chaperone that promotes the maturation, structural maintenance and proper regulation of specific target proteins involved i.e. in cell cycle control and signal transduction
	Heat shock protein 90-beta		gi|194378142	130	
2	Ezrin		gi|11276938	110	• Involved in connections of major cytoskeletal structures to the plasma membrane • In epithelial cells, required for the formation of microvilli and membrane ruffles on the apical pole
3	KHSRP protein		gi|54648253	145	• Role in mRNA trafficking • Gene expression activation
4	Heat shock protein 75		gi|2865466	100	• Involved in maintaining mitochondrial function and polarization • Negative regulator of mitochondrial respiration able to modulate the balance between oxidative phosphorylation and aerobic glycolysis
6	TATA-binding protein-associated factor 2N isoform 2		gi|4507353	52	• RNA and ssDNA-binding protein with roles during transcription initiation at distinct promoters
7	Alpha-tubulin		gi|340021	232	• Tubulin is the major constituent of microtubules
8	Pyrroline-5-carboxylate dehydrogenase		gi|1353248	81	• Irreversible conversion of delta-1-pyrroline-5-carboxylate (P5C), derived either from proline or ornithine, to glutamate
	UDP-glucose 6-dehydrogenase isoform 1		gi|4507813	72	• Involved in the biosynthesis of glycosaminoglycans
10	Translation initiation factor 4A–III		gi|496902	144	• Core component of the splicing-dependent multiprotein exon junction complex • mRNA processing • mRNA splicing • mRNA transport • Nonsense-mediated mRNA decay • RNA processing • Translation regulation
11	Glutamate dehydrogenase 1, mitochondrial precursor		gi|4885281	64	• Cellular amino acid biosynthetic process • Converts L-glutamate into alpha-ketoglutarate
12	Alpha-enolase isoform 1		gi|4503571	119	• Multifunctional enzyme that, as well as its role in glycolysis, plays a part in various processes such as growth control, hypoxia tolerance and allergic responses
14	Laminin-binding protein		gi|34234	170	• Required for the assembly and/or stability of the 40 S ribosomal subunit • Also functions as a cell surface receptor for laminin • Plays a role in cell adhesion to the basement membrane and in the consequent activation of signaling transduction pathways
16	Keratin 10		gi|28317	51	• Structural protein which forms the intermediate filament
17	Heat shock protein 27		gi|35182	124	• Involved in stress resistance and actin organization • Negative regulation of apoptotic process • Positive regulation of angiogenesis • Positive regulation of blood vessel endothelial cell migration
	**Proteins with ≥2 times lower expression in MCF-7 cells treated with cyclopamine + tamoxifen compared with control cells**				
**No**	**Protein Description**		**GI Accession**	**Score**	**General Functions**
18	far upstream element-binding protein 1		gi|17402900	172	• Regulates MYC expression
19	far upstream element-binding protein 1		gi|17402900	172	• Regulates MYC expression
20	Heterogeneous nuclear ribonucleoprotein H		gi|5031753	116	• Component of the heterogeneous nuclear ribonucleoprotein (hnRNP) complexes which provide the substrate for the processing events that pre-mRNAs undergo before becoming functional • pre-mRNA alternative splicing regulation
21	Elongation factor 1 alpha		gi|31092	40	• Promotes the GTP-dependent binding of aminoacyl-tRNA to the A-site of ribosomes during protein biosynthesis
22	Tu translation elongation factor, mitochondrial, isoform CRA_b		gi|119572383	148	• Promotes the GTP-dependent binding of aminoacyl-tRNA to the A-site of ribosomes during protein biosynthesis
23	C protein		gi|306875	97	• Protein C is a vitamin K-dependent serine protease that regulates blood coagulation by inactivating factors Va and VIIIa in the presence of calcium ions and phospholipids • negative regulation of apoptotic process • post-translational protein modification
26	Triosephosphate isomerase		gi|136066	75	• carbohydrate metabolic process
	**2-D gel of MCF-7 cells treated with cyclopamine + tamoxifen**				
**No**	**Protein Description**	**GI Accession**		**Score**	**General Functions**
28	GRP78 precursor, partial	gi|386758		133	• Involved in the correct folding of proteins and degradation of misfolded proteins • Cellular protein metabolic process • Cellular response to antibiotic • Cellular response to glucose starvation • Negative regulation of apoptotic process • Positive regulation of cell migration
29	Heat shock protein 27	gi|662841		91	• Involved in stress resistance and actin organization • Negative regulation of apoptotic process • Positive regulation of angiogenesis • Positive regulation of blood vessel endothelial cell migration
	**Proteins with ≥2 times higher expression in MCF-7 cells treated with cyclopamine + tamoxifen compared with control cells**				
**No**	**Protein Description**	**GI Accession**		**Score**	**General Functions**
31	Keratin 8, isoform CRA_a	gi|119617057		76	• Plays a role in maintaining cellular structural integrity and also functions in signal transduction and cellular differentiation
32	Prohibitin isoform 1	gi|4505773		308	• Role in human cellular senescence and tumor suppression • Antiproliferative activity is reported to be localized to the 3′ UTR • Positive regulation of cell proliferation and migration

General Functions are obtained from the UniProt and NCBI Gene databases. Protein numbers correspond to the numbers marked on the 2-D gels (Figure S1). Numbers in the table correspond to spot numbers denoted on the 2-D gel images; missing numbers in the table are unidentified proteins or proteins with score less than 39.

### Shh regulates ERα expression in MCF-7, but not SkBr-3 cell line

**Figure 5:**
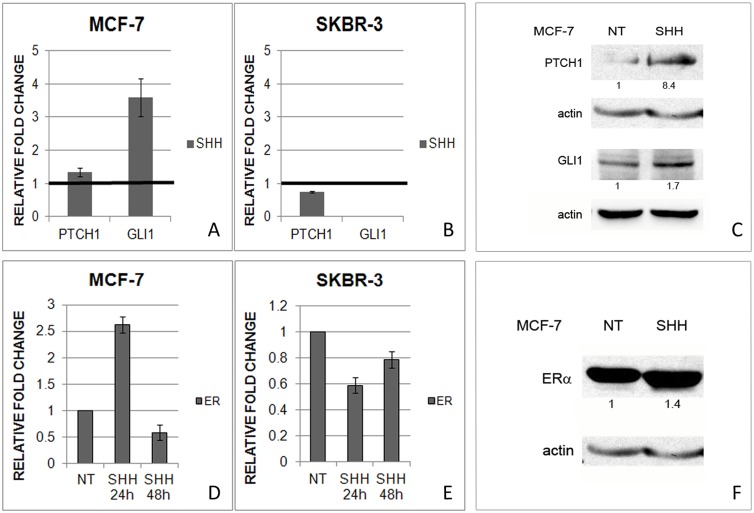
Effect of stimulation with Shh protein on pathway activity in MCF-7 (A,C) and SkBr-3 cells (B). Gene expression levels are shown on graph as relative fold change relative to non-treated conditions with reference value 1 pointed out with emboldened bar. Relative gene expression of *ERα* after treatment with Shh protein (D,E). Non-treated cells (NT) have a relative value 1. ERα protein expression in MCF-7 cells increases after treatment with Shh protein for 48 h (F) Protein bands were quantified and normalized relative to actin and non-treated conditions and the relative values are denoted below each band.

Since inhibition of ERα with tamoxifen affected Hh-Gli signaling we wanted to establish whether there is cross-talk between these two pathways. Therefore, both cell lines were treated with Shh protein. MCF-7 cells responded to stimulation with exogenous Shh protein by Hh-Gli pathway activation (Fig. 5A, C) whereas the ER-negative cell line did not respond to Shh stimulation (Fig. 5B). Interestingly, short-term Shh treatment also had an effect on ERα expression in ER-positive cell line, which was increased (Fig. 5D, F), but this effect was relatively quickly negated 48 h post-treatment (Fig. 5D). In the SkBr-3 cell line there was no upregulation of *ERα* in response to Shh protein, but rather a slight downregulation (Fig. 5E).

**Figure 6:**
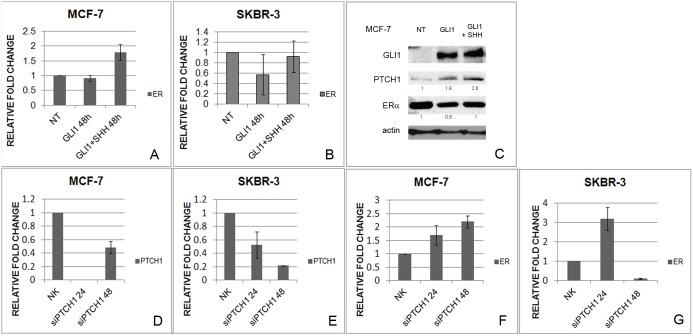
Gene and protein expression levels after transfection with *GLI1* (GLI1) and additional stimulation with Shh protein (GLI1+SHH). *ERα* gene expression increases in MCF-7 cells only after additional Shh stimulation (A) while *ERα* gene expression does not change in SkBr-3 cells (B). Gli1, Ptch1 and ERα protein levels in MCF-7 cells after *GLI1* transfection and additional Shh stimulation (C). Protein bands were quantified and normalized relative to actin and non-treated conditions and the relative values are denoted below each band. Relative gene expression of *PTCH1* (D, E) and *ERα* (F,G) after silencing of *PTCH1* gene in MCF-7 and SkBr-3 cell line. Efficient silencing (<30% of residual expression) was achieved 24 h post-transfection in MCF-7 cell line, and 48 h post-transfection in SkBr-3 cell line.

To check whether the effect of Shh on *ERα* is mediated via the canonical Hh-Gli signal transduction, cells were transfected with *GLI1*. After transfection and additional Shh stimulation, Gli1 and Ptch1 gene and protein expressions were elevated in MCF-7 cells (Fig. 6C, S2 Figure), whereas *ERα* was upregulated in MCF-7 cell line only after exogenous Shh stimulation (Fig. 6A). On the protein level ERα expression decreased after *GLI1* transfection, but an increase was visible after Shh addition, compared with only transfected cells (Fig. 6C). This suggests that ERα regulation is not mediated transcriptionally via Gli1 transcription factor, but rather directly by Shh protein.

Even though the transfection was successful in SkBr-3 cells, shown by upregulation of *GLI1* and *PTCH1* expression (S2 Figure), it had no effect on *ERα* gene expression which was expected since there is only a low basal level of *ERα* mRNA expression and no ERα protein production in these cells (Fig. 6B).

To confirm a direct impact of Shh protein on *ERα* we silenced *PTCH1*, the primary Shh receptor, which would cause an increase in free, unbound Shh protein that could in turn interact with ERα and increase its activity. The effect was induction of *ERα* expression in MCF-7 cells, suggesting Shh protein has a direct effect on ERα. (Fig. 6D, F) For SkBr-3 cell line, sufficient knockdown of *PTCH1* was achieved 48 h post-transfection (Fig. 6E) and the effect on *ERα* was downregulation of gene expression (Fig. 6G).

### Shh protein interacts with ERα

**Figure 7:**
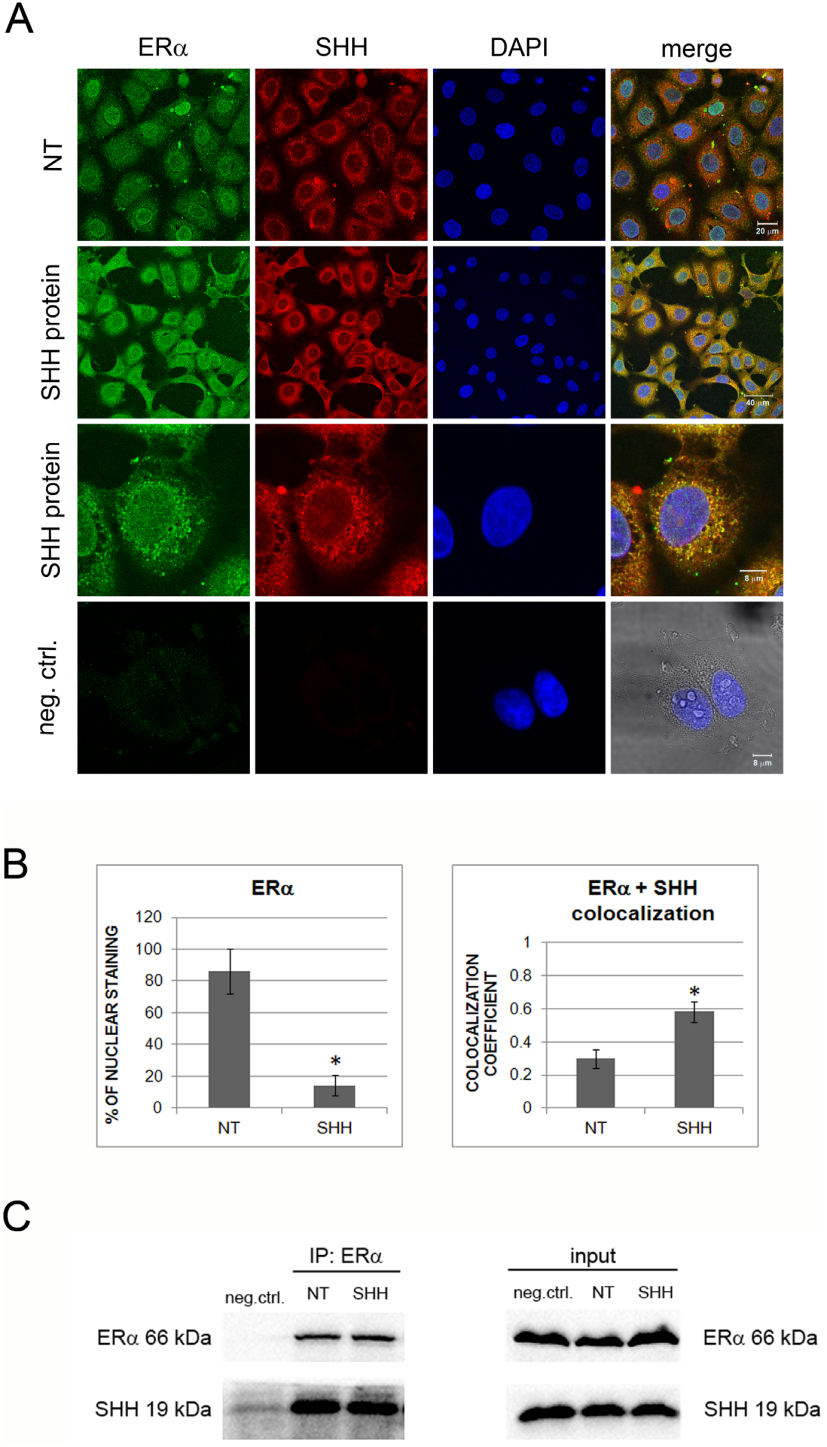
Immunofluorescent staining of MCF-7 cell line in non-treated cells (NT) and treated with Shh protein detected by confocal microscopy. ERα is stained green (column 1), Shh is stained red (column 2), nuclei are stained blue with DAPI (column 3), and the last column shows the overlay of signals. Yellow staining shows areas of green and red signal co-localization (A). Shh-treated cells show significantly decreased nuclear staining and increased co-localization of ERα and Shh compared to non-treated cells, as determined by ImageJ software, (*) P<0.05. (B). Shh protein co-immunoprecipitates with ERα protein in MCF-7 cells, both in non-treated conditions and after treatment with exogenous Shh protein for 48 h; NT = non-treated, neg.ctrl. = negative control. Western blot of input proteins is provided as control for presence of the proteins in cell lysates (C).

To verify whether Shh has a direct effect on ERα, cells were treated with Shh protein, for 48 h and localization of Shh and ERα was visualized. Non-treated cells showed Shh staining in a granular pattern in the cytoplasm, mostly surrounding the nucleus, while ERα was scattered in the cytoplasm and stronger in the nuclei. Shh treatment caused an interesting effect: co-localization of Shh and ERα in the cytoplasm of the cells (Fig. 7A). There was very little co-localization of ERα and Shh in untreated cells, but after 48 h-treatment with Shh protein there is significantly less nuclear staining of ERα (P = 0,0003) and ERα and Shh co-localized in the cytoplasm (P<0.0001) (Fig. 7B). This suggests that Shh acts directly on ERα, modifying its activity. Co-immunoprecipitation results however, indicate an interaction of Shh and ERα proteins in general, regardless of treatment with exogenous Shh protein (Fig. 7C). This is not unusual as the MCF-7 cells produce high amounts of Shh protein. These results undoubtedly show an interaction between Shh and ERα proteins, which is the first mention of direct interaction between these two proteins. However, adding exogenous Shh protein did not increase this interaction, as would be expected from the immunofluorescence data. It is possible that, since the MCF-7 cells already produce high amounts of Shh protein, addition of exogenous protein has no influence on the interaction rate. However, the fact that there is an obvious interaction between these two proteins is a new and intriguing finding that needs to be investigated further as it opens new possibilities in the aspect of Hh-Gli signaling research in ER-positive breast cancer.

## Discussion

The role of Hh-Gli signaling in breast cancer is still unclear, especially regarding their association with steroid receptor signaling. To date the findings of Hh-Gli component expression in breast cancer cell lines is contradictory, particularly for Shh and Gli1. We found expression of Gli1 and Shh in the ER-positive cell line (MCF-7), but Ramaswamy et al. on the other hand found no expression of Shh in MCF-7 cells [21]. This inconsistency may be due to the fact that the authors looked only at the expression of unprocessed Shh protein (45 kDa). This is supported by the expression of *SHH* at the mRNA level which they did find. Two other studies, on the other hand, did find Shh expression in MCF-7 cells [7], [37]. Also, some studies show high expression of *GLI1* in ER-negative cell lines, including SkBr-3 [20], [38], but in our hands *GLI1* expression was not detectable in SkBr-3 cells. Recently a study showed a positive correlation between *ERα* and *GLI1* expression [39], supporting lower levels of *GLI1* in the ER-negative cell line. Even though these authors did find very low *GLI1* expression in SkBr-3 it was much lower than in MCF-7. Given the lower levels of *GLI1* in MCF-7 cells that we detected it is not surprising it was undetectable in SkBr-3.Cyclopamine has been tested together with gefitinib in prostate cancer cell lines, where the combined treatment induced a supra-additive inhibitory growth effect on serum-free and serum-stimulated cell lines. This effect is established through cell cycle arrest in G1 phase and increased apoptosis. Cyclopamine and gefitinib-treated cells showed a decreased ability for invasion, and this effect was amplified in combined treatment [40]. In other studies on prostate cancer cells cyclopamine used in combination with ErbB inhibitors gefitinib or lapatinib showed a synergistic effect [41], [42] and combination of docetaxel+cyclopamine+gefitinib induced more intensive cell death compared to either treatment alone [43]. In cholangiocarcinoma treatment with cyclopamine and MEK inhibitor U0126 showed an additive effect, especially in cells with *KRAS* mutation [44].

Our results regarding the effect of cyclopamine on breast cancer cells are in agreement with previous studies that have shown that cyclopamine inhibits human breast cancer cell growth by increased apoptosis [19]. In a study by Che et al. [22] cyclopamine was reported to have anti-proliferative, anti-invasive and anti-estrogenic potency in human breast cancer. This is similar to our findings which also showed the anti-estrogenic effect of cyclopamine, ERα gene expression was downregulated after cyclopamine treatment.

In the ER-positive breast cancer cell line, however, combined treatment with cyclopamine and tamoxifen increased cell viability after short-term treatment, but it was not seen in ER-negative cells. This effect was dose-dependent, and competition experiments have shown that higher concentrations of both compounds are required for the survival effect. Short-term combined treatment of MCF-7 cells upregulated the Hh-Gli signaling pathway and promoted cell migration (Figs. 2–4).

To elucidate the effect of the combination of these two drugs on the profile of expressed proteins we performed proteomic profiling of cells treated with a combination of cyclopamine and tamoxifen as well as control non-treated cells. This analysis revealed that a small but unique set of proteins is upregulated upon combination treatment in comparison with non-treated cells. All of them have been linked to cell proliferation and migration (Table 1). GRP78, a known survival factor, has been known to mediate signaling pathways that lead to proliferation an migration [33], [34]. Prohibitin was initially shown to block cell proliferation [45], but this ability was attributed to its 3′ untranslated region [46]. However, there is emerging evidence that prohibitin as a protein is required for cell proliferation and adhesion [47]. This protein is also known for activating the Raf-MEK-ERK signaling pathway and inducing cell migration [36], [48]. Another protein found to be upregulated after treatment with cyclopamine and tamoxifen is keratin 8. The data on the role of keratin 8 in cancer are inconsistent. Some studies show that keratin 8 overexpression correlates with lower tumorigenicity, invasiveness and motility [49], while others found it to be correlated with poor prognosis, invasiveness and cell migration [35], [50], [51]. HSP27, which is expressed under stressful conditions, is found both in treated cells and non-treated cells, but the protein was shifted in relation to the protein in non-treated cells suggesting it was modified. It has been found that the phosphorylated form of this protein participates in stress resistance and act as a negative regulator of apoptosis and a positive regulator of proliferation and migration [52]–[55]. This suggests that a combination of these drugs potentially enhances the migration ability of these cells, which is consistent with the results obtained by the wound healing and transwell migration assays, showing that cells treated with the combination of drugs have a higher migration capacity than the non-treated ones. Whether this effect is related to the upregulation of the Hh-Gli signaling pathway remains to be investigated. It should be looked into whether the Hh-Gli signaling pathway can directly or indirectly affect the expression of these proteins.

Apart from Hh-Gli pathway being regulated by compounds affecting ERα (tamoxifen), the communication works also in the other direction, from Hh-Gli signaling to ERα. The link between ERα and Hh-Gli signaling pathways has been addressed in previous studies. It was shown that upregulation of ERα by E2 also upregulated Shh which canonically activated Hh-Gli signaling and Gli1 expression in human breast cancer cells [37]. The same link was observed in ERα positive gastric cancer [56]. In both studies the vice versa link was not observed. We on the other hand, show a potential mechanism of ERα regulation through Hh-Gli signaling. Although there may be a transcriptional link between Hh-Gli and estrogen signaling via FoxM1 [25], [57], this does not seem to be the case here. Transfection of *GLI1* does not automatically induce transcription of *ERα*, like it does of *PTCH1*; suggesting *ERα* expression is not regulated transcriptionally via Gli1. Only after exogenous addition of Shh protein there is an induction in *ERα*, regardless of *GLI1* levels. Our co-immunoprecipitation assay confirmed a direct link between Shh and ERα proteins (Figs. 5–7). It is possible that the cholesterol modification of the Shh protein plays a role in this interaction since cholesterol is the precursor molecule for steroid hormones, but this remains to be analyzed. This interaction may be the cause of upregulation of ERα activity and consequently upregulation of ERα gene and protein expression. Silencing of *PTCH1* leads to a reduced number of receptor molecules on the membrane, allowing increased binding of endogenous Shh to the *ERα*, which leads to upregulation of *ERα* expression (Fig. 6), since ERα autoregulates its own expression [58].

The mechanism which is responsible for the increased viability of ER-positive cell line after combined treatment with cyclopamine and tamoxifen, in comparison with either treatment alone, is not clear. We show that the Hh-Gli signaling is upregulated and proteins involved in proliferation and migration enhancement are expressed, but the link between them and the Hh-Gli signaling remains to be elucidated. Although Hh-Gli signaling seems to be a good potential target for breast cancer therapy, caution must be advised, especially when combining therapies. We have demonstrated that combined treatment of cyclopamine and tamoxifen may induce an opposite effect, providing cells with short-term survival and increased ability to migrate, which may be deleterious for the patient. On the other hand, we show a potential direct link between Shh and ERα proteins. According to our results Shh can bind ERα and activate it. This might be a mechanism that enhances survival of breast cancer cells with expression of Shh, even in estrogen deficient conditions.

## Supporting Information

S1 Figure **2-D gels of non-treated control MCF-7 cells (A) and MCF-7 cells treated with cyclopamine and tamoxifen (B).** 2-D gel of MCF-7 cells treated with a combination of cyclopamine and tamoxifen with indicated spots that have ≥2 times higher expression compared with control cells (C). 2-D gel of MCF-7 cells treated with a combination of cyclopamine and tamoxifen with indicated spots that have ≥2 times lower expression compared with control cells (D). Indicated spots were used for further MS analysis. Results are shown in Table 1.
(TIF)

S2 Figure ***GLI1*** **and** ***PTCH1*** **gene expression levels after transfection with GLI1 plasmid in ER-positive MCF-7 cells (A, C) and ER-negative SkBr-3 cells (B, D).**
(TIF)
