# Supplementary figures and images for: Combination of Cyclopamine and Tamoxifen Promotes Survival and Migration of MCF-7 Breast Cancer Cells – Interaction of Hedgehog-Gli and Estrogen Receptor Signaling Pathways
Source: PLoS One. 2014 Dec 12;9(12):e114510. doi: 10.1371/journal.pone.0114510 (PMC4264763; doi:10.1371/journal.pone.0114510)

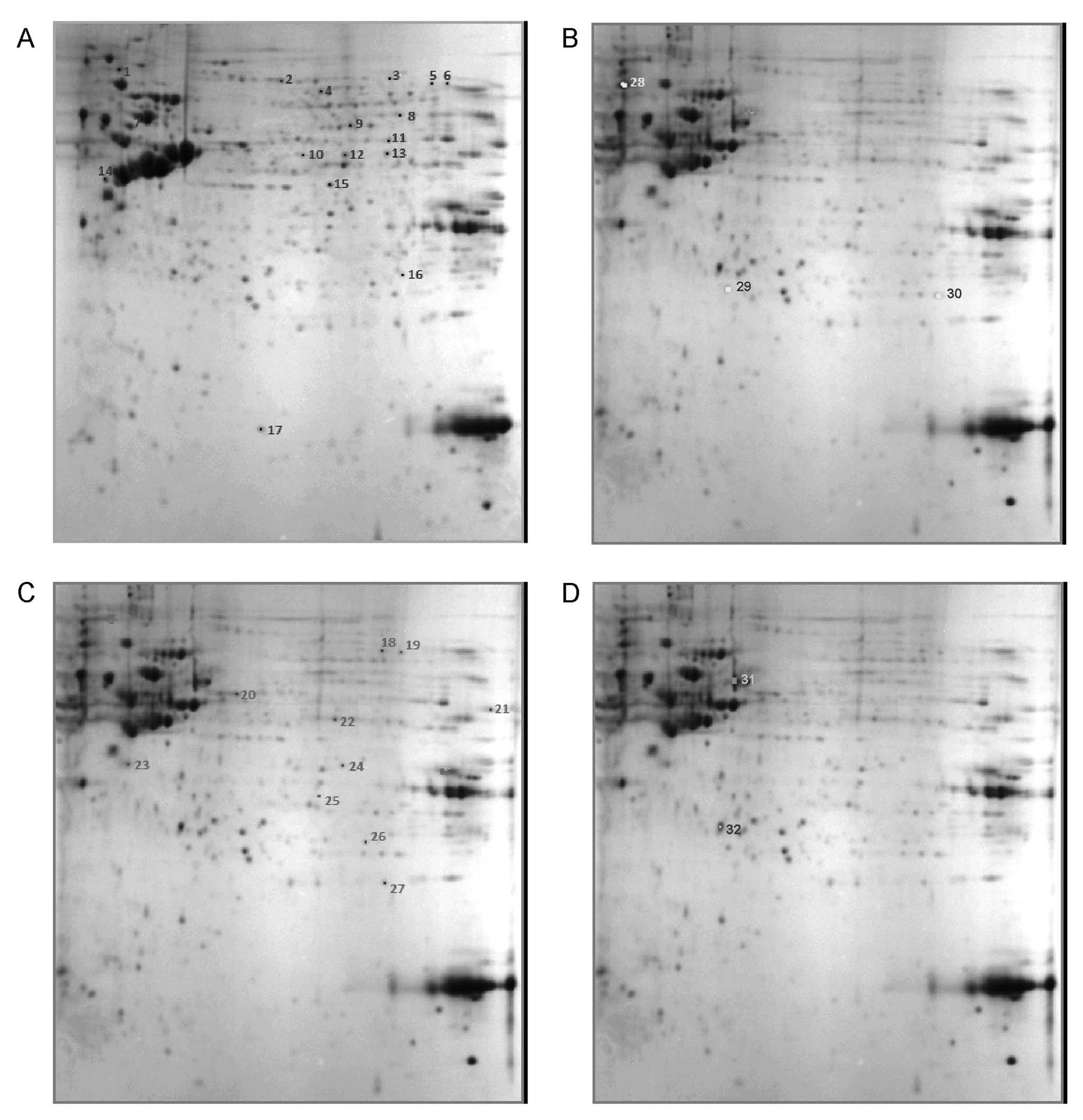

Supplement: S1 Figure — 2-D gels of non-treated control MCF-7 cells (A) and MCF-7 cells treated with cyclopamine and tamoxifen (B). 2-D gel of MCF-7 cells treated with a combination of cyclopamine and tamoxifen with indicated spots that have ≥2 times higher expression compared with control cells (C). 2-D gel of MCF-7 cells treated with a combination of cyclopamine and tamoxifen with indicated spots that have ≥2 times lower expression compared with control cells (D). Indicated spots were used for further MS analysis. Results are shown in Table 1. (TIF) [file pone.0114510.s001.tif]

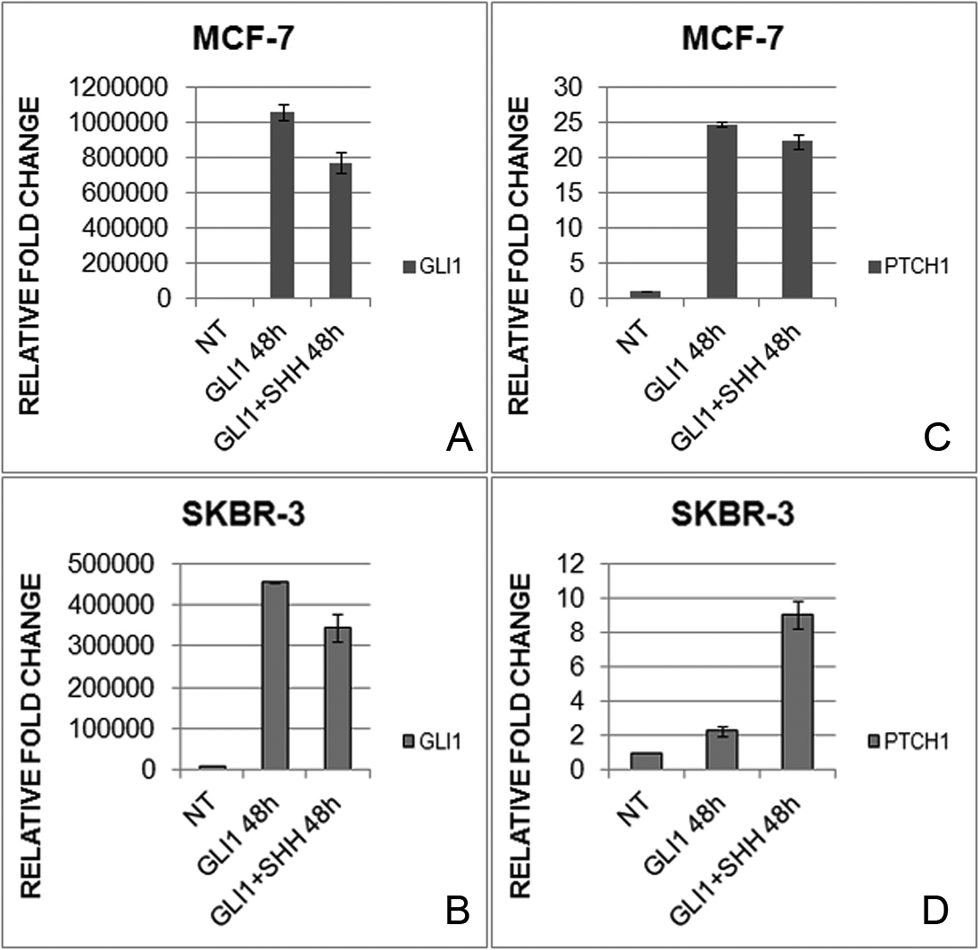

Supplement: S2 Figure — GLI1 and PTCH1 gene expression levels after transfection with GLI1 plasmid in ER-positive MCF-7 cells (A, C) and ER-negative SkBr-3 cells (B, D). (TIF) [file pone.0114510.s002.tif]
